# “How Do We Actually Do Convergence” for Disaster Resilience? Cases from Australia and the United States

**DOI:** 10.1007/s13753-021-00340-y

**Published:** 2021-03-25

**Authors:** Shefali Juneja Lakhina, Elaina J. Sutley, Jay Wilson

**Affiliations:** 1 Wonder Labs, San Jose, CA 95128 USA; 2 Civil, Environmental and Architectural Engineering, University of Kansas, Lawrence, KS 66045 USA; 3 Clackamas County Disaster Management, Oregon City, OR 97045 USA

**Keywords:** Convergence research, Disaster resilience, Disaster risk reduction, Research ethics

## Abstract

In recent years there has been an increasing emphasis on achieving convergence in disaster research, policy, and programs to reduce disaster losses and enhance social well-being. However, there remain considerable gaps in understanding “how do we actually do convergence?” In this article, we present three case studies from across geographies—New South Wales in Australia, and North Carolina and Oregon in the United States; and sectors of work—community, environmental, and urban resilience, to critically examine what convergence entails and how it can enable diverse disciplines, people, and institutions to reduce vulnerability to systemic risks in the twenty-first century. We identify key successes, challenges, and barriers to convergence. We build on current discussions around the need for convergence research to be problem-focused and solutions-based, by also considering the need to approach convergence as ethic, method, and outcome. We reflect on how convergence can be approached as an ethic that motivates a higher order alignment on “why” we come together; as a method that foregrounds “how” we come together in inclusive ways; and as an outcome that highlights “what” must be done to successfully translate research findings into the policy and public domains.

## Introduction

Since 2005, a number of national and international policies have articulated why the grand challenge of our times will be to reduce vulnerability to social, economic, and environmental disasters (National Science and Technology Council 2005; United Nations 2009, 2015; National Research Council 2012). There is now wide agreement that reducing vulnerability to disasters will require bringing together diverse forms of knowledge, from across disciplines, geographies, and scales (Spangler 1982; Tierney 2005, 2014; Greenberg and Lowrie 2011; National Research Council 2012, 2014; Aitsi-Selmi et al. 2016; Kendra et al. 2019; Peek et al. 2020). However, due to persistent disciplinary silos and associated methodological challenges, this integration has largely not happened and is still being called for in the literature (Davidson 2015; Esnard and Lai 2018; Mitsova 2018; Reilly et al. 2018; Peek et al. 2020). Further, there remain ongoing ethical and methodological debates about how to achieve such integration in ways that are inclusive, respectful, and empowering (Gaillard and Peek 2019; Gaillard et al. 2019). In this context, we want to examine how achieving convergence in disaster research, policy, and programs can lead to inclusive, just, and equitable outcomes. Distinct from multidisciplinary collaboration, which is still conducted in disciplinary silos, we are interested in examining how greater convergence in disaster research, policy, and programs can enable new ways of bringing together knowledge, practices, and insights in interdisciplinary and transdisciplinary ways, to contribute to the development of novel and inclusive methods, tools, and outcomes.

For over a decade, there has been an increasing emphasis on achieving convergence across disciplinary efforts in the domains of science, technology, and society (Roco and Bainbridge 2003, 2013; National Research Council 2014; Roco 2016). Convergence was initially explored as a means to motivate innovation and discovery in medicine, engineering, and robotics (Roco and Bainbridge 2003). During a first level of convergence, in the late 1990s and early 2000s, the development of nanotechnology enabled a deep convergence of various disciplines, resulting in new understanding of how materials, devices, and living systems interact. In the 2000s, a second level of convergence brought together nanotechnology, biotechnology, information technology, and the cognitive sciences, resulting in the integration of a range of emerging technologies. The third and current level of convergence, since 2010 and beyond, is enabling an integration of human knowledge, behavior, and technologies to respond to societal values and needs (Roco and Bainbridge 2013).

In 2016, convergence was adopted as one of 10 Big Ideas for future investments^1^ by the United States’ National Science Foundation (NSF). The NSF defines convergence research as a “means of solving vexing problems, in particular, complex problems focusing on societal needs” (NSF 2018). Under its Growing Convergence Research (GCR)^2^ priority, the NSF now requires research projects to demonstrate convergence—characterized by “deep integration across disciplines” and “novel solutions to pressing societal needs” (NSF 2018). Recognizing the relevance of convergence in disaster research, in 2018, NSF awarded a grant to the Natural Hazards Center at the University of Colorado in Boulder, to establish CONVERGE—a five-year initiative to establish and strengthen multidisciplinary networks in the United States and to grow natural hazards convergence research. In July 2019, the center adopted convergence as the theme for its 44th Annual Natural Hazards Research and Applications Workshop,^3^ convened in Broomfield, Colorado, to deliberate on how convergence can be undertaken as a collaborative process of crossing borders and boundaries to identify and solve complex problems in novel ways (for a summary, see Peek 2019). In closing the workshop, the center’s director, Lori Peek motivated us to reflect on “how do we actually do convergence?^4^” In the following section, we reflect on key points and case studies presented during our panel discussion at the 2019 workshop, to examine how convergence can be achieved for inclusive, just, and equitable outcomes across geographies and scales.

Convergence thinking is of course not new to disaster risk reduction and resilience efforts. At least since the 1950s, disaster researchers, emergency managers, and communities have been coming together to work on disaster risk management problems (Mostafavi and Ganapati 2019). In the past decades, convergence has also been pursued in international policy frameworks for disaster risk reduction including climate change adaptation and the equitable achievement of the sustainable development goals (Aitsi-Selmi et al. 2016; Shaw et al. 2016) although gaps remain in how this momentum translates to local-level efforts and outcomes (Olson et al. 2020). In disaster studies, Fritz and Mathewson (1957, p. 1) were the first to define convergence as “the mass movement of people, messages, and supplies toward the disaster struck area,” which they characterized as: personal, informational, and material convergence, respectively. Over the decades, researchers have added to Fritz and Mathewson’s typology to include convergence behavior observed in new kinds of crisis scenarios (see Kendra and Wachtendorf 2003) and to reflect emerging modes of crisis communication (see Subba and Bui 2017). Most recently, Peek et al. (2020) have put forth a definition and framework for convergence research to be more widely adopted in disaster studies. They define convergence research as:

An approach to knowledge production and action that involves diverse teams working together in novel ways—transcending disciplinary and organizational boundaries—to address vexing social, economic, environmental and technical challenges in an effort to reduce disaster losses and promote collective well-being (Peek et al. 2020, p. 2).

We seek to extend Peek et al.’s (2020) definition to examine how convergence can be framed in “whole of society” terms to include local institutions and communities working alongside disaster researchers to address systemic risks in just, inclusive, and equitable ways. In doing so, we explore pathways to adopting a pluralistic approach that can bring together diverse forms of knowledge—disciplinary, institutional, traditional, and phenomenological, including people’s lived experiences of rapidly changing landscapes in the twenty-first century.

In the following section, we build on Peek et al.’s (2020) calls for convergence research to be problem-focused and solutions-based, by also reflecting on how convergence can be understood as ethic, method, and outcome. We consider how convergence can be approached as an ethic that motivates a higher order alignment on “why” we come together—across disciplines, scales, and geographies, with diverse people, communities, and institutions; as a method that foregrounds “how” we come together in inclusive ways to formulate research and collaborative projects that address ongoing processes of disaster risk creation; and as an outcome that highlights “what” must be done to successfully translate findings from the physical and social sciences into the policy and public domains at a time when disaster risk science is highly politicized or altogether ignored (Lakhina 2019).

## Cases of Convergence in Action

In July 2019, the 44th Natural Hazards Research and Applications Workshop in Broomfield, Colorado, brought us together for a panel discussion—Cases of Convergence in Action—to examine how convergence can be achieved in disaster risk reduction and resilience initiatives. We discussed cases of convergence in action from diverse geographies—New South Wales in Australia, and North Carolina and Oregon in the United States; and across sectors of work—community, environmental, and urban resilience. Our panel discussion was guided by the following five questions put forth by our moderator^5^:

1. How can convergence produce outcomes that are more than the sum of individual efforts?
2. How is convergence different in practice from collaboration?
3. What factors enable active convergence?
4. What factors undermine or constrain convergence?
5. What is the role of leadership in creating pathways for convergence?

Below, we provide some brief context on each case study. We then attempt to answer the above-stated questions to show how we interpreted and achieved convergence through our respective initiatives, while also identifying remaining challenges. The first case demonstrates how local institutions and services can achieve inclusive and empowering outcomes through sustained engagement and partnership with communities. The second case highlights the need to build trusting relationships and interdisciplinary understanding in project teams and with communities. The third case identifies the urgent need and entry points for adopting a convergence approach to address systemic risks. The three cases also enable reflection on why a convergence approach is especially relevant to examining the long-haul and cascading impacts of the ongoing COVID-19 pandemic.

### Converging with Collaboration, Accountability, Responsiveness, and Empowerment (CARE) in New South Wales, Australia

As part of the lead author’s doctoral research at the University of Wollongong, Australia (2016−2019), a collaborative research project was undertaken in 2017, in partnership with city councils, multicultural agencies, emergency services, community-based organizations, and people from diverse refugee backgrounds living across the Illawarra region of New South Wales, Australia (Lakhina 2018a, 2018b, 2019; Lakhina et al. 2019). The Illawarra is a coastal region about 40 miles south of Sydney, encompassing the three local government areas of Wollongong, Shellharbour, and Kiama. Situated between an escarpment to the west and the South Pacific Ocean to the east, the Illawarra experiences a range of natural hazards, including bushfires, heatwaves, storms, flash flooding, and lightning. The region’s fast diversifying demographics and changing land use patterns are likely to result in increasing exposure of people and infrastructure to future climate change impacts.

The Illawarra case contributes at least three ways of achieving convergence in disaster research, policy, and programs. First, convergence can be understood as an ethic that motivates a higher order alignment on why we come together to work across silos with diverse people, communities, and institutions. Convergence requires a caring approach that takes a long and deep view of places, people, histories, and politics by opening constructive dialogue around future pathways (see Goldstein et al. 2014). By adopting a person-centered approach to co-learning disaster resilience with people from diverse backgrounds, the Illawarra case demonstrates a pathway to converging with CARE—collaboration, accountability, responsiveness, and empowerment (Lakhina 2019). The CARE approach emphasizes:

*collaboration* with people and institutions, holding the research team (ideally, comprising researcher/s, research participants, and local institutions) *accountable* to commonly defined research objectives and ethics, ensuring *responsiveness* to people’s needs and challenges, while sustaining *empowering* forms of engagement and partnerships (Lakhina 2019, p. 94).

In adopting an ethic of CARE, the Illawarra project centered community perspectives and regularly translated findings for institutional stakeholders. For example, during a workshop in November 2017, six thematic focus group discussions were facilitated between community and institutional representatives. These discussions enabled service providers, institutions, and community members to solve complex problems together, while developing a clear understanding of mutual capacities and needs. However, this kind of discussion required institutional representatives to look past their traditional top-down methods of “teaching” community members about disaster resilience, and instead, be willing to deeply engage with and learn from community perspectives, questions, and experiences. The workshop discussions led to the development of the New South Wales State Emergency Services’ first Multicultural Liaison Unit, comprising liaison officers from diverse refugee backgrounds (see Lakhina et al. 2019). Formed in December 2017, this unit now serves as a model for not just informing newly arrived people but also systematically engaging and partnering with culturally and linguistically diverse populations across the Illawarra on an ongoing basis (Ellis 2018; University of Wollongong 2018). The development of this unit corrects previously top-down approaches of disseminating information to emerging communities by adopting a sustained practice of engaging and partnering with emerging communities from refugee backgrounds. This outcome demonstrates how local institutions and services can reach beyond their historical silos and come together in novel ways to engage and partner with emerging communities for more just, equitable, and inclusive disaster resilience outcomes (Lakhina 2018a, 2019; Lakhina et al. 2019).

**Fig. 1:**
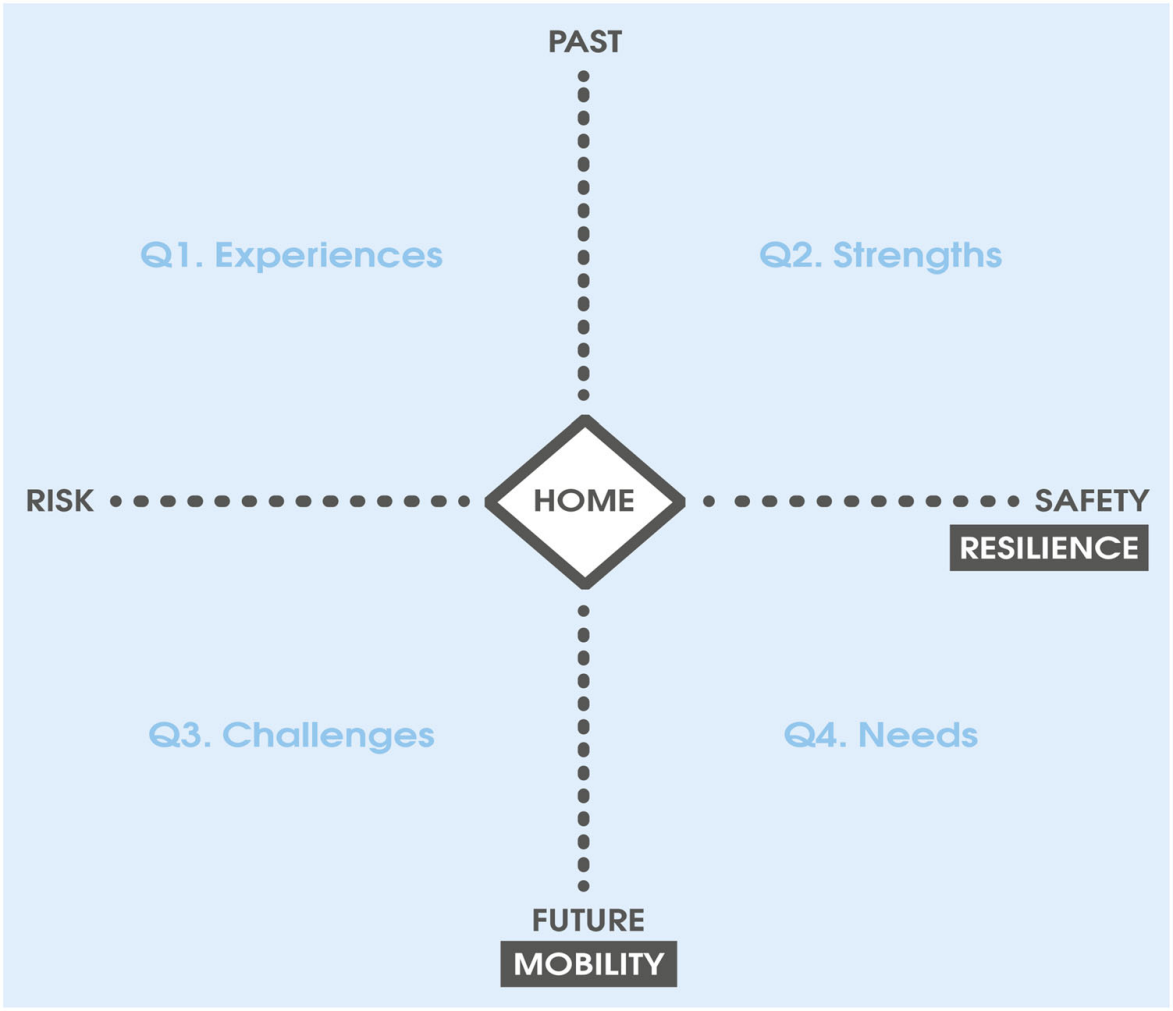
Resilience narrative map template

Second, local and cultural contexts can play an important role in how we converge. Convergence calls for greater recognition and engagement with the everyday micropolitics of places, institutions, and people. This entails listening closely and engaging deeply with people’s lived experiences and everyday practices of safety and well-being. For example, adopting a person-centered methodology and tool—the resilience narrative map (see Fig. 1)—allowed project partners to engage with culturally and geographically diverse narratives and practices, thereby developing a more inclusive understanding of what constitutes disaster resilience for newly arrived people in the Illawarra. As community experiences (in Q1), perceived strengths (in Q2), challenges (in Q3), and remaining needs (in Q4), were plotted in the resilience narrative mapping template (Fig. 1), local institutions and services could better visualize the kinds of community capacities that remain unengaged, and particular needs that remain unaddressed among new and emerging communities in the Illawarra (see Lakhina (2018a) for examples from Burma, the Democratic Republic of Congo, Iran, Iraq, Liberia, Syria, and Uganda). In this way, convergence can be understood as a method that foregrounds how we come together in inclusive ways to reduce vulnerability and enhance social well-being.

Third, convergence in outcomes can highlight what must be done to successfully translate research findings into policy and public actions for just, inclusive, and equitable outcomes. Convergence can entail new partnerships, interfaces, and ways of coming together. Convergence can also lead to divergence and transformations that challenge and disrupt existing structures and standards. For example, the process of coming together in the Illawarra revealed a lack of program coordination between the local emergency services, and the settlement and multicultural services, community-based organizations, places of worship, and community volunteers (see Lakhina (2018a, 2018b) for more about the project’s key findings). Addressing this coordination gap had implications for how local agencies come together to serve newly arrived people from refugee backgrounds in ways that are collaborative, accountable, responsive, and empowering. In this way, convergence can also reveal new and unforeseen entry points by challenging conventional ways of thinking and doing things. Undertaking a process of convergence entails an emphasis on “why” and “how” while “what” becomes contingent on the local and institutional context. Such open-endedness reveals new ways of doing things by thinking creatively about future pathways and modes of engagement. This can be difficult in situations where the power balance is likely to get upset but committing to a moral vision of “why” and “how” emphasizes the need to let go of conventional ways of thinking and practicing inclusion in disaster resilience work.

The Illawarra case also shows why it is important to diversify our understanding of leadership. Institutions and disciplinary “experts” are not the only ones who display leadership. Forms of leadership can also be observed in people’s daily activism, advocacy, and caring relationships within communities. Such leadership is amply demonstrated in the narratives of the Illawarra’s community, particularly by women and mothers from refugee backgrounds. The role of leadership in convergence approaches should not only be emphasized as one of “expertise” in a particular subject or domain, but also as the ability to sustain trust and care in relationships—healing relationships across political divides, bridging institutional relationships across silos, and fostering personal relationships between people and communities.

**Fig. 2:**
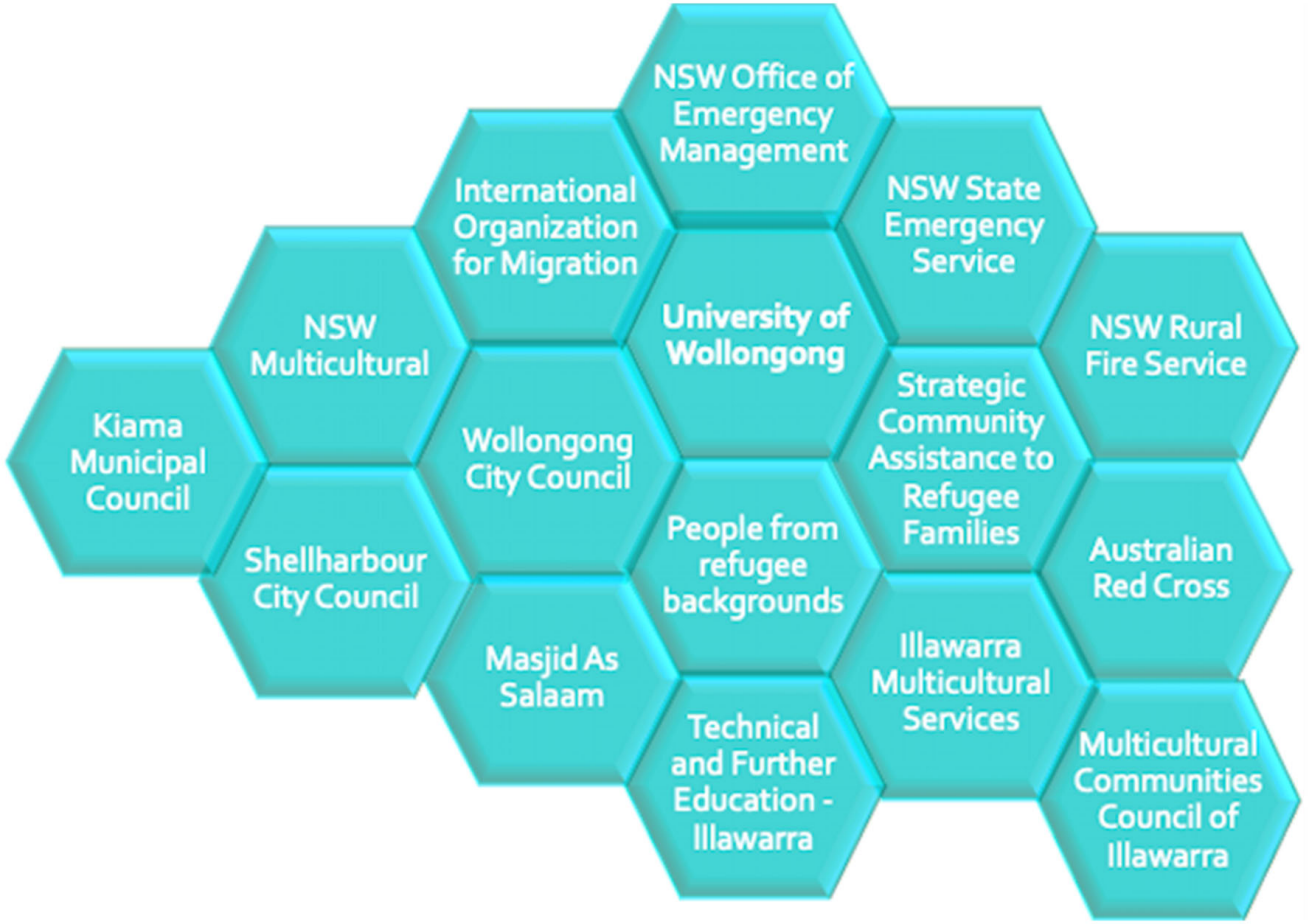
Converging with collaboration, accountability, responsiveness, and empowerment (CARE) in the Illawarra case

Building on this insight, the Illawarra project developed a framework for “co-learning disaster resilience”—a sustained process for informing, engaging, and partnering with newly arrived and recently settled people from refugee backgrounds (Lakhina 2018a). Understood in this way, co-learning is not a top-down or bottom-up approach. Instead, it represents a honeycomb of relationships (see Fig. 2), which can organically develop over time, through a coming together of efforts, a bringing together of diverse perspectives, and a continuous process of learning with and from one another. The honeycomb presented here is an attempt to provide an alternative visualization to network-based approaches that tend to represent power relations between points, hubs, and nodes in systems.

A deeper commitment to an ethic of CARE can also guide ongoing ethical and methodological discussions about a code of ethics for disaster research (Gaillard and Peek 2019; Gaillard et al. 2019). Perhaps giving greater recognition to the ethics and practices of how we converge can provide concrete pathways for reimagining disaster resilience as a set of diverse, transformative, and intercultural practices. Extending learning from the Illawarra case, how can disaster research, policy, and programs embrace convergence as an opportunity to move from an emphasis on efficiency to care, diagnosis to dialogue, and policy prescriptions to co-learning with people’s diverse experiences and perspectives (Lakhina 2019)?

### Community Resilience Planning in North Carolina, United States

**Fig. 3:**
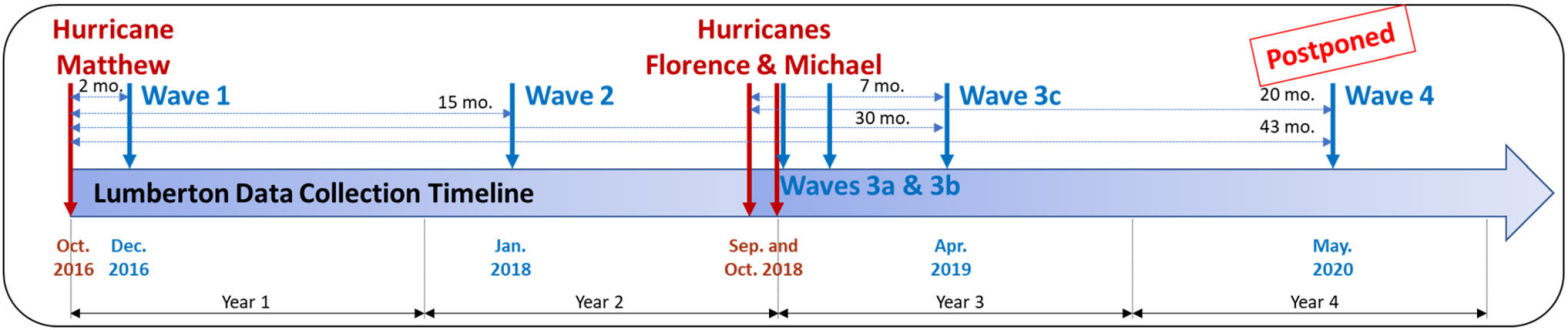
Data collection timeline designed to capture impact, disruption, recovery, and decision-making processes without interfering with emergency operations

The Center for Risk-Based Community Resilience Planning, funded by the National Institute of Standards and Technology (NIST), has approximately 100 team members spanning diverse disciplines, such as engineering, computer science, economics, sociology, and urban planning, and representing academic, industry, and government backgrounds. The project’s goal is to produce a web-based application for modeling and assessing community-level resilience.^6^ To collect the necessary data to support and/or validate ongoing resilience algorithms, the second author leads a longitudinal field study effort in Lumberton, North Carolina for the center. Diverse communities across the City of Lumberton in Robeson County were affected by two major hurricanes within a period of 23 months: Hurricane Matthew in October 2016 and Hurricane Florence in September 2018. The scope of the field study has centered on understanding how different community sectors, namely housing, business, local schools, and local and state governments, are impacted by disaster, and go through recovery both as individual and connected sectors. The field study team has collected data five times between 2016 and 2019 (see Fig. 3 for a field study timeline). Data collection for Wave 4 has been postponed due to the COVID-19 pandemic (see van de Lindt et al. (2020) and Sutley et al. (2021) for more information). For each data collection exercise, close-ended surveys are sent to the same housing units and businesses, and semistructured interviews are conducted with the same school and local government focal points.

The North Carolina case also contributes at least three ways of achieving convergence in disaster resilience initiatives. First, guided by a convergence ethic, the field study was designed to better reflect community impact, resilience, and recovery, to emphasize why we must come together to study disasters. The field study team was composed of structural engineers to examine building and infrastructure damage, sociologists to examine how the floodwaters impacted and disrupted households and businesses, and urban planners to examine how the city was responding to the disruption. Prior to deployment in 2016, the sociologists and urban planners were cross-trained in measuring flood damage, and the structural engineers were cross-trained in human subjects’ research, administering surveys, and conducting interviews. Early in the instrument development phase, multidisciplinary teams were formed to ensure the information being captured was well-rounded. Each wave of data collection consisted of more than 20 researchers from different disciplines. In the field, teams of two, three, or four researchers, spanning disciplinary expertise, administered the household surveys together. While extra training and time was required for instrument development, it was only through bringing these disciplines together that a richer picture of resilience and recovery could be formulated. The multidisciplinary, multisector, longitudinal study design became increasingly important amid the COVID-19 pandemic to document the cascading impacts of ongoing flood recovery amid a public health crisis, such as new parental educational responsibilities, lost employment, new business interruption, and housing instability.

**Fig. 4:**
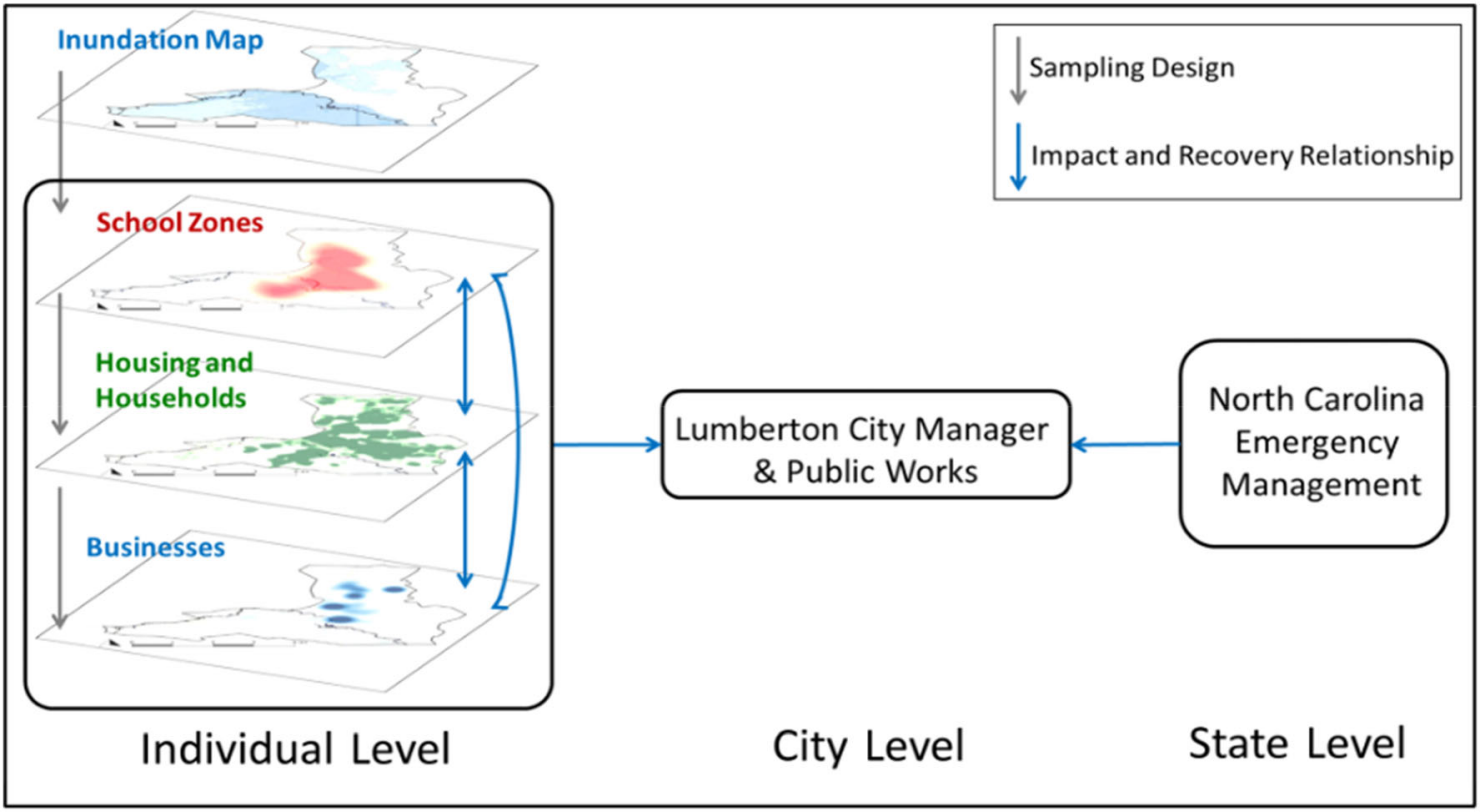
Research design consisted of a multilevel data collection process that enabled a more holistic understanding of the disaster and enabled a feedback loop between the community and the research team

Second, the project experience shows why local and cultural contexts can play an important role in what data we collect, and how we collect and use data for convergence. For example, the Lumber River was the source of flooding after both Hurricane Matthew in 2016 and Hurricane Florence in 2018. There were apparent trends in neighborhood locations, where more minority and low-income households lived south of the river in the floodplain, and white and wealthier households lived north of the river. This socio-geographical context was considered in the sampling approach and used as dependent variables in subsequent analysis (Sutley et al. 2019; Watson et al. 2020; van de Lindt et al. 2020). Additionally, the smallest income category reported by the United States Census and the American Housing Survey is USD 5000. However, more than a third of Lumberton’s population lives below the national poverty line, so the housing survey instrument included income categories as low as USD 1000. Furthermore, the culture in Lumberton is largely religious, and specifically, Christian. While households were more likely to be at home on weekends, Sunday mornings and lunch times were not used for data collection to respect associated religious practices. Finally, the people of Lumberton were severely, but differentially, impacted by both flood disasters. When we approached households and businesses for surveys, our team passed out information sheets on local mental health services, and shared information on where federal aid offices were set up. As depicted in Fig. 4, the team surveyed households and businesses to understand broader and individual experiences of disruption and recovery, and also interviewed representatives from the school systems, civic works departments, city, and state to understand the multilevel impact of the disaster. The latter enabled shared learning between our team and the community, and served to establish a feedback loop with decision makers to share our cross-sector research findings.

Third, regular face-to-face meetings over the span of a longitudinal project that lasted more than five years enabled the team to develop trusting relationships and interdisciplinary understanding. This experience provides important insights on how we converge for solutions-based outcomes, as identified by Peek et al. (2020). On the data collection side, the longitudinal nature of the study was critical to develop a more holistic understanding of recovery processes. In each subsequent wave, the same samples were revisited to document the recovery process. The same team members would be sent back, as best as possible, to the same locations over the years, to help build a trusting relationship between the project team and the people of Lumberton. The trust and relationships we cultivated with the people of Lumberton will be evermore critical for the success of virtual data collection in Wave 4.

In terms of the development of our project team, the Center for Risk-Based Community Resilience Planning was funded in February 2015 but it was not until October 2016 that the first field study was executed. The year and a half lead time for team development, including learning interdisciplinary terminology, and building trust, was crucial to our successful field-based collaboration. The center hosts a semiannual meeting where all team members meet in person; additional meetings are organized for subsets of working groups, including at shared conferences and partner institutions. Between in-person meetings, weekly video conference calls have kept the team working closely together and humanized the research process. These meetings have taken many forms, including information updates, working sessions, and presentations from individuals to learn more about their background. Leadership, particularly in academia, can sometimes hamper convergence by putting up barriers that protect traditional disciplinary boundaries. In presenting our project’s experience we show how leadership can instead begin to emphasize the need for interdisciplinary and transdisciplinary approaches to solve real-world problems. Through the project’s longitudinal collaborative effort, all team members learned from each other and helped to facilitate convergence research. This convergence outcome has been especially valuable for early career scholars and collaborators who took on more substantial roles, thereby substantively contributing to interdisciplinary disaster scholarship.

### Critical Energy Infrastructure (CEI) Hub in Portland, Oregon, United States

**Fig. 5:**
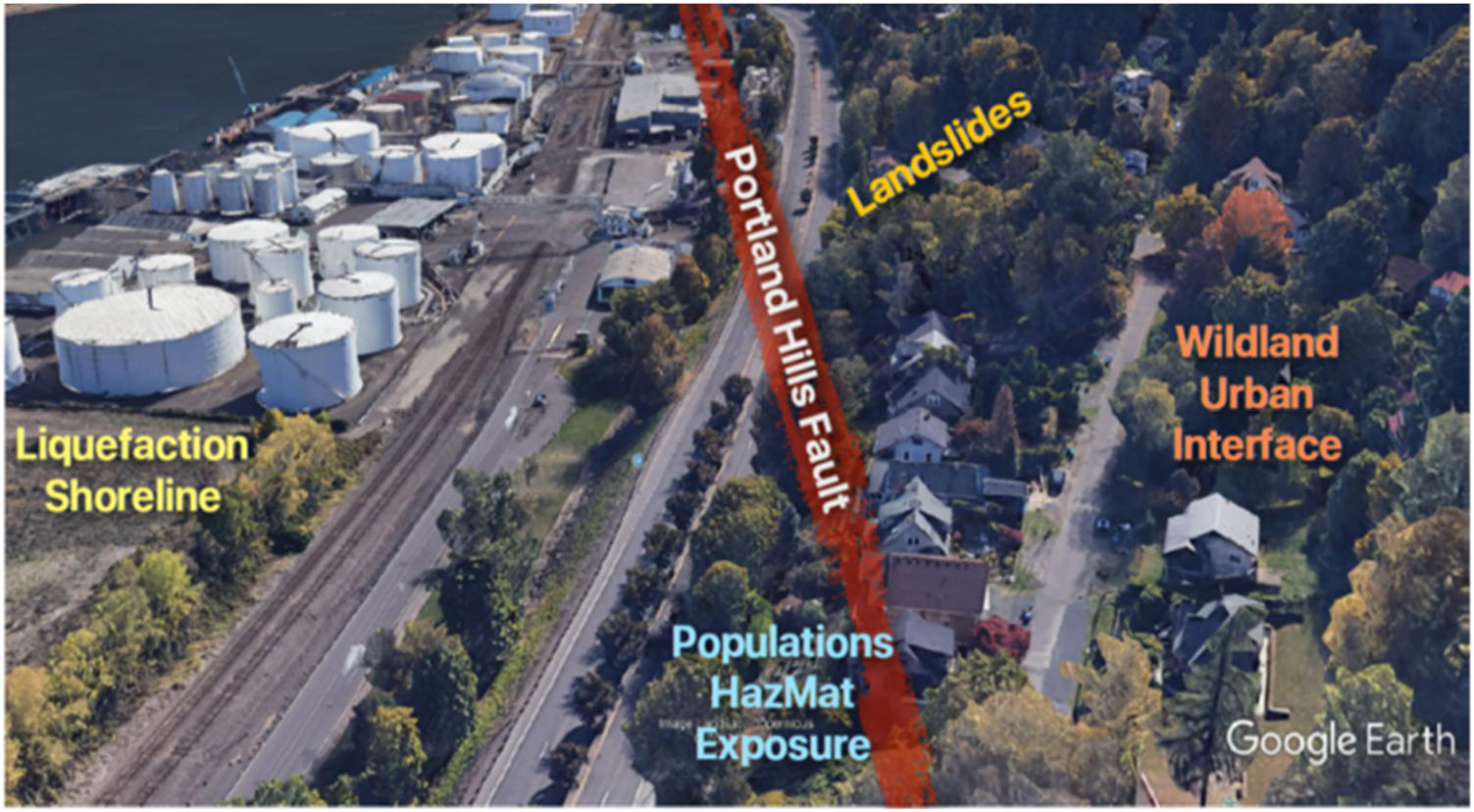
Critical Energy Infrastructure (CEI) Hub hazard exposure

Oregon’s Critical Energy Infrastructure (CEI) Hub in Portland holds most of the state’s liquid petroleum. A number of facilities located in the CEI Hub are nearly 100 years old and highly vulnerable to seismic, wildfire, and landslide hazards (DOGAMI 2012; Portland State University 2019). The six-mile corridor of petroleum storage tanks, fuel pipelines, and related energy infrastructure are located along the lower Willamette River shoreline immediately upstream of the confluence with the Columbia River. Due to a high concentration of natural hazards and associated risks, the CEI (Fig. 5) is considered to be Oregon’s “Achilles’ heel.”

Adopting a convergence approach, as ethic, method, and outcome, to address this disaster risk is urgent because the potential social, environmental, and economic impacts are likely to be enormous (Wilson 2019). Seismic research since the 1980s has shown that the City of Portland is at high risk of earthquakes and associated hazards (Flynn et al. 1999). Approaches to seismic mitigation have emphasized strengthening oil storage infrastructure (OSSPAC 2019). However, aligning seismic policies with climate change policies and finding common ground to understand and reduce community vulnerabilities has presented a major challenge in past years. A possible convergence solution will require reducing dependency on oil and promoting sustainable and clean energy approaches for future generations (Portland Bureau of Planning and Sustainability 2019). This will entail reducing exposure to at least five known hazards in this area.

First, the six miles of riverside shoreline is a high liquefaction hazard area with poor alluvial soils underlying over 500 tanks. These tanks date back to the 1920s; most were built in the 1950s and 1960s with no seismic design standards. A 2019 study by the City of Portland and Portland State University determined that the average daily capacity of the tank farm is 360 million gallons of petroleum products (OSSPAC 2019). While most tanks have containment berms to protect from accidental release, the tanks and berms would be subject to ground failure from liquefaction or lateral spreading during an earthquake and would create a cascading failure for a large percentage of the facilities’ tanks and pipelines.

Second, the CEI Hub sits along the Portland Hills fault, which has the potential to produce up to a magnitude 6.8 crustal earthquake and result in severe to violent ground shaking for the immediate area. While the recurrence period of earthquakes associated with this fault is 1,000 years, the most likely earthquake scenario is a magnitude 9.0 on the Cascadia subduction zone, that has a 500-year return interval and the last one occurred 320 years ago (OSSPAC 2013). A magnitude 9.0 earthquake would generate a strong and long period of shaking. There are currently no building codes or standards to prepare structures for this type of seismic event. The impacts of an earthquake, particularly a large earthquake, in this area will be further exacerbated by the vulnerability of decades old structures full of liquid petroleum.

Third, the CEI Hub and the west Portland Hills are prone to landslides and debris flows. A large number of these historic slides are documented by the Oregon Department of Geology and Mineral Industries (DOGAMI) and occur regularly with heavy and prolonged rainfall. A worst case scenario would be a damaging earthquake during the rainy season that triggers multiple landslides. These slides could directly impact numerous facilities in the CEI Hub, but also impair access for emergency response. A fourth natural hazard on the west side of the Willamette River is wildfire. Portland’s Forest Park is one of the largest urban forests in the country and is nestled in the west Portland Hills. During red flag periods, Forest Park and the intermix neighborhoods could easily become a conflagration from an ignition source in the CEI Hub and vice versa (see Miller 2020). The concentration of hundreds of petroleum storage tanks, along with tanks of ammonia and chlorine, present an imminent risk for the adjacent neighborhoods on both sides of the Willamette River. Smoke plumes and toxic chemical releases in the air and water would rapidly threaten the thousands of residents in short proximity from the tank farms.

Finally, any major release from the CEI Hub due to berm failures during an earthquake would generate a catastrophic oil spill with no emergency capacity to contain or mitigate spread. Such a spill on the lower Willamette River would pour into the lower Columbia River and reach the Pacific Ocean within days. A release of only 3% of the oil volume would equal the 1989 Exxon Valdez oil spill of 10.8 million gallons and would qualify as a spill of national significance, resulting in chronic and long-term impacts on trade and exports (Pacific Northwest Waterways Association 2019), wildlife, natural resources, water intakes, tourism, fishing, and general liveability, especially for tribal communities.

**Fig. 6:**
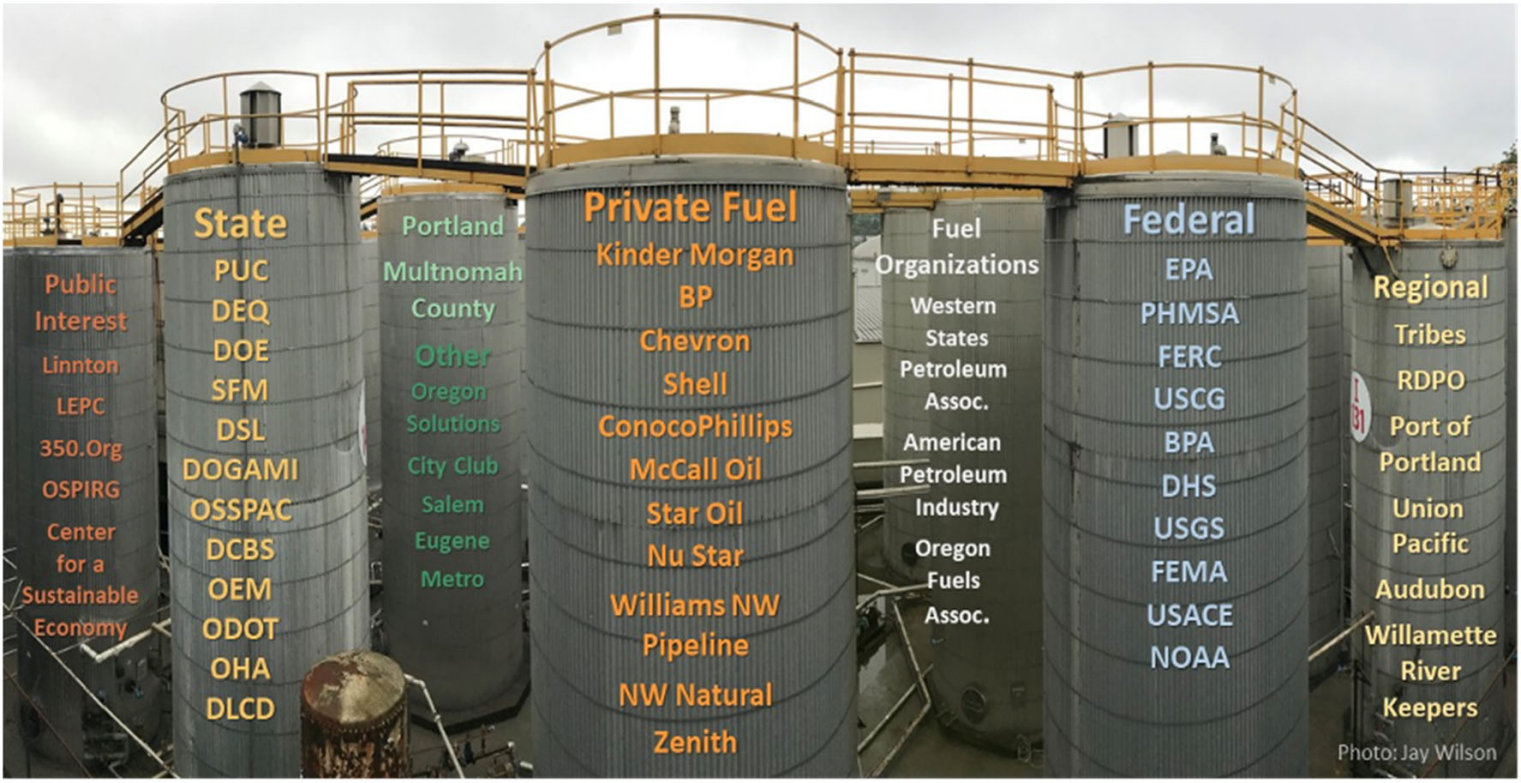
Critical Energy Infrastructure (CEI) Hub: Stakeholder silos. Acronyms listing (left to right): LEPC, Local Emergency Planning Committee; OSPIRG, Oregon State Public Interest Research Group; PUC, Public Utilities Commission; DEQ, Department of Environmental Quality; DOE, Oregon Department of Education; SFM, State Fire Marshall; DSL, Oregon Department of State Lands; DOGAMI, Oregon Department of Geology and Mineral Industries; OSSPAC, Oregon Seismic Safety Policy Advisory Commission; DCBS, Oregon Department of Consumer and Business Services; OEM, Office of Emergency Management; ODOT, Oregon Department of Transportation; OHA, Oregon Health Authority; OLCD, Department of Land Conservation and Development; EPA, Environmental Protection Agency; PHMSA, Pipeline and Hazardous Materials Safety Administration; FERC, Federal Energy Regulatory Commission; USCG, United States Coastal Guard; BPA, Bonneville Power Administration; DHS, United States Department of Homeland Security; USGS, United States Geological Survey; FEMA, Federal Emergency Management Agency; USACE, United States Army Corps of Engineers; NOAA, National Oceanic and Atmospheric Administration; RDPO, Regional Disaster Preparedness Organization

These compound risks entail the probable loss of nearly all of Oregon’s critical fuel immediately following a damaging earthquake (DOGAMI 2012; OSSPAC 2013). Further, the long-term impacts of infrastructure disruptions can be manifold, including on public health, local economies, and the environment (Chang 2016). Unfortunately, the potential of this cascading environmental and economic disaster, while very likely in a foreseeable earthquake, has not been studied, planned for, or widely communicated, to the extent required. In response to these dire scenarios, disaster simulation exercises have underscored the need for emergency fuel resources, along with fuel allocation planning, and studies for possible alternative sites and technical mitigation for existing structures. In 2015, Portland City Council passed a resolution to oppose the expansion of fossil-fuel dependent infrastructure; in 2016, a zoning ordinance prohibited the building of new fossil-fuel storage facilities; and in 2019, the state’s Governor signed legislation requiring that rail companies develop oil spill response plans (Cunningham 2019). A recent policy (State of Oregon 2018) listed the CEI Hub among the Governor’s top seismic risk priorities and two related CEI Hub reports (Oregon Solutions (2019) and OSSPAC (2013)) addressed the administrative and policy struggles of working with oil companies and regulatory agencies to find an agreeable solution. However, progress has been elusive. Figure 6 illustrates the political separations and restrictive lines of authority that continue to fragment responsibility and obstruct any meaningful progress.

This case highlights the complexity of tackling transdisciplinary issues and the urgent need for implementing a convergence-based approach that aligns seismic standards with climate change adaptation policies, and leverages recovery-informed planning towards a sustainable future. Specifically, Portland’s case highlights the need for convergence research, policy, and programs to deeply understand the environmental, social, and economic costs and consequences of transitioning to clean energy. It underlines the need for more regulatory oversight and coordination at the local, state, and national levels to bridge divides between oil companies and regulatory agencies. It also highlights the need for sustained ethical leadership to undertake inclusive visioning exercises and promote resilience values for what socially just recovery will entail in the event of cascading disaster impacts. As the first Pacific Northwest generation to understand the magnitude 9.0 seismic risk and global climate risks, our children and grandchildren need not inherit the same post-war era infrastructure that has already outlived its life expectancy and is operating well over its design capacity. The ongoing COVID-19 pandemic and calls for social justice further reiterate the need for convergence approaches that can address Portland’s systemic vulnerability to cascading disasters. The urgent need of the hour is for a coherent national climate strategy to outline locally enforceable targets for reducing emissions, adopting clean energy fuel, and strengthening public infrastructure, while addressing social inequities through a convergence approach.

## Conclusion

In recent years there has been an increasing emphasis on achieving convergence in disaster research (Peek et al. 2020), policy, and programs (Olson et al. 2020). However, there remain considerable gaps in understanding “how do we actually do convergence?” What does convergence entail and how can it enable diverse disciplines, people, and institutions to address systemic risks in the twenty-first century? In this article, we argue that the process of coming together can no longer be left to happenstance, or the leadership of a few individuals and institutions. Instead, we present three case studies to concretely show how convergence can be approached as ethic, method, and outcome, across different kinds of disaster research, policy, and program efforts.

The three empirical cases we present here do not represent a comprehensive or final solution. Instead, we seek to reflect on key successes, challenges, and barriers to convergence. The first case, from the Illawarra region of New South Wales in Australia, demonstrates how local institutions and services can work towards facilitating more inclusive, just, and empowering resilience outcomes by developing modes of sustained engagement and partnership with emerging communities. Adopting a person-centered approach to co-learning disaster resilience with people from diverse backgrounds, the case demonstrates a pathway to converging with CARE—collaboration, accountability, responsiveness, and empowerment (Lakhina 2018a, 2018b, 2019; Lakhina et al. 2019). The case enables reflection on how convergence can respond to some of the ethical and methodological dilemmas currently experienced in disaster research and practice (Gaillard and Peek 2019; Kendra et al. 2019). The second case, from North Carolina, United States, highlights the need for research programs to have enough time to build trusting relationships, and develop interdisciplinary and transdisciplinary understanding across project teams. The case also shows how we do convergence in project design and team interactions can have real implications for how we interact with vulnerable populations. The third case identifies the challenges of aligning policies for mitigating seismic risk and climate change to achieve convergence for clean energy in Portland, Oregon. It highlights the daunting challenges cities can face in planning for systemic risks and mobilizing public preparedness for cascading disaster impacts. While we do not identify any convergence trends underway in Oregon, the case study underlines the urgent need to adopt convergence as ethic, method, and outcome, to prevent catastrophic loss from imminent disasters.

Through these three case studies we explore how achieving greater convergence in disaster risk reduction and climate adaptation initiatives can lead to more inclusive, just, and equitable futures. We hope our insights can motivate reflection among similar ongoing efforts in disaster research, policy, and programs. The need for such reflection is particularly important in the context of the global COVID-19 pandemic. While researchers, institutions, and community-based organizations have, to a varying extent, adapted to a virtual world in the past year, it remains to be seen how novel modes of engagement and partnership can be developed with communities during the ongoing global pandemic. We are hopeful that convergence principles can guide us in the task ahead. The pandemic has shown us the need for human connection and compassion, and the urgency of reaching across disciplinary silos, bringing together diverse expertise, and responding to the unfolding crisis in novel, caring, and thoughtful ways.

Looking to the future, we recommend approaching convergence in at least three ways: (1) as an “ethic” that motivates a higher order alignment on “why” we come together, across disciplines, scales, and geographies, with diverse people, communities, and institutions; (2) as a “method” that foregrounds “how” we come together in inclusive ways to formulate research and collaborative projects that address ongoing processes of disaster risk creation; and (3) as an “outcome” that highlights “what” must be done to successfully translate findings from the physical and social sciences into the policy and public domains at a time when disaster risk science is highly politicized or altogether ignored.

Addressing systemic risks will require disaster researchers and managers to approach convergence in the “whole of society” domain. Opening up the convergence framework in this way will be important to enable the inclusion of diverse forms of knowledge with implications for how disaster researchers frame problems and co-develop solutions with local institutions and communities on the frontline of mounting disaster impacts.

## Footnotes

1 https://www.nsf.gov/news/special_reports/big_ideas/.

2 https://www.nsf.gov/pubs/2019/nsf19551/nsf19551.htm.

3 https://hazards.colorado.edu/workshop/2019.

4 https://hazards.colorado.edu/news/director/closing-comments-from-the-2019-natural-hazards-workshop.

5 Balachandran, B. Personal email communication, 31 May 2019.

6 See http://resilience.colostate.edu to learn more about the project.
